# Poly (Vinylidene Fluoride-Hexafluoropropylene)–Lithium Titanium Aluminum Phosphate-Based Gel Polymer Electrolytes Synthesized by Immersion Precipitation for High-Performance Lithium Metal Batteries

**DOI:** 10.3390/gels10030179

**Published:** 2024-03-04

**Authors:** Xuanan Lu, Jianguo Luo, Lingxiao Lan, Bing Zhang, Zhikun Chen, Yujiang Wang, Xinghua Liang, Qinglie Mo

**Affiliations:** 1 Guangxi Key Laboratory of Automobile Components and Vehicle Technology, Guangxi University of Science & Technology, Liuzhou 545006, China; a15007751851@163.com (X.L.); 18677215099@163.com (J.L.); 13152528815@163.com (Y.W.); lxh304@126.com (X.L.); 100001683@gxust.edu.cn (Q.M.); 2 Liuzhou Wuling Automobile Industry Co., Ltd., Liuzhou 545006, China; 3 Foshan Taoyuan Advanced Manufacturing Research Institute, Foshan 528225, China; 13067887972@163.com

**Keywords:** gel polymer electrolytes, excellent rate performance, the immersion precipitation method, LATP

## Abstract

Gel polymer electrolytes (GPEs) have high safety and excellent electrochemical performance, so applying GPEs in lithium batteries has received much attention. However, their poor lithium ion transfer number, cycling stability, and low room temperature ionic conductivity seriously affect the utilization of gel polymer electrolytes. This paper successfully synthesized flexible poly (vinylidene fluoride-hexafluoropropylene)–lithium titanium aluminum phosphate (PVDF-HFP-LATP) gel polymer electrolytes using the immersion precipitation method. The resulting GPE has a porous honeycomb structure, which ensures that the GPE has sufficient space to store the liquid electrolyte. The GPE has a high ionic conductivity of 1.03 ×10^−3^ S cm^−1^ at room temperature (25 °C). The GPE was applied to LiFePO_4_/GPE/Li batteries with good rate performance at room temperature. The discharge specific capacity of 1C was as high as 121.5 mAh/g, and the capacity retention rate was 94.0% after 300 cycles. These results indicate that PVDF-HFP-LATP-based GPEs have the advantage of simplifying the production process and can improve the utility of gel polymer lithium metal batteries.

## 1. Introduction

Due to the large consumption of traditional energy and the deterioration of the environment, the development of renewable clean energy and its technological breakthrough are imminent. For this reason, various energy conversion and storage devices have been rapidly developed. Lithium metal batteries (LMBs) are one of the more prominent energy storage devices and are considered an ideal anode due to their unprecedented theoretical capacity (3860 mAh g^−1^) and extremely low redox potential (3.04 V compared to standard hydrogen electrodes). Meanwhile, lithium metal batteries have a high operating voltage, a long cycle life, and a high specific discharge capacity, so they are widely used in automobiles and large-scale energy storage devices [1,2,3]. Conventional liquid lithium metal batteries contain a large amount of organic electrolyte, which can lead to electrolyte leakage and other safety hazards [4,5,6,7]. Solid-state lithium metal batteries can solve the above safety problems [8]. Many researchers have studied various solid-state electrolytes (SSEs) in depth [9,10]. However, SSEs have low ionic conductivity. SSEs are in point-to-point contact with the positive and negative electrodes of the battery, resulting in a high interfacial impedance. Therefore, the performance of solid lithium metal batteries is not as good as that of liquid lithium metal batteries [11,12,13]. Gel polymer electrolytes (GPE) have the characteristics of low fluidity, good flame retardancy, and interfacial compatibility, which can combine the advantages of both liquid electrolytes and polymer electrolytes. Replacing the liquid electrolyte with a polymer gel electrolyte is considered to be an effective solution to the current problems faced by LMBs.

GPEs include a polymer backbone and a liquid organic electrolyte. The commonly used polymers are polyethylene oxide (PEO) [14,15], poly(vinylidene fluoride-co-hexafluoropropylene) (PVDF-HFP) [16], polymethyl methacrylate (PMMA) [17], and polypropylene eyelet (PAN) [18]. Among them, PVDF-HFP is a promising material. Featuring a high dielectric constant, it achieves exceptional room temperature ionic conductivity, superior mechanical strength, and electrochemical stability. However, the high crystallinity of PVDF-HFP leads to unsatisfactory performance of the gel polymer electrolyte formed from this material. To improve the performance of GPEs, researchers have added a certain amount of inorganic filler to GPEs. Ceramic fillers can be divided into inert fillers and active inorganic electrolyte fillers. Compared with inert inorganic fillers such as SiO_2_ and BaTiO_3_, active inorganic electrolyte fillers can not only increase the amorphous area of the polymer matrix, making it easier to transport lithium ions, but can also provide an additional diffusion path for Li^+^, thus improving the electrochemical performance more effectively. Active inorganic electrolyte fillers Li_0.33_La_0.557_TiO_3_ (LLTO), Li_7_La_3_Zr_2_O_12_ (LLZO), Li_10_GeP_2_S_12_ (LGPS), and Li_1.7_Al_0.3_Ti_1.7_(PO_3_)_4_ (LATP) are well-known electrolyte materials. The NASICON-type fast ionic conductor LATP is one of the best room-temperature conductive electrolytes discovered so far among the oxide ionic fast conductors. After the polymer matrix was incorporated with LATP, the amorphous region of the polymer matrix increased, which is more beneficial for lithium ion transport. LATP provides an additional diffusion pathway for lithium ions. Due to its stability and wider electrochemical stabilization window, many researchers are interested in it [19]. 

Due to the rapid development of the lithium metal battery industry, the electrolyte membrane is the most critical part of solid-state batteries, which is currently prepared mainly by the thermotropic phase separation (TPMS) method. However, the electrolyte prepared this way absorbs less per unit volume, resulting in a lower GPE ionic conductivity [20]. The immersion precipitation (IP) method has great potential due to its easy preparation process and the large number of porous membranes that can be obtained [21].

This work presents a simple and feasible strategy and process method to fabricate gel electrolytes with excellent performance. PVDF-HFP-LATP gel electrolytes were prepared using the immersion precipitation method. By manipulating the dosage of LATP, we could fabricate polymer electrolytes possessing exceptional electrochemical characteristics and remarkable liquid absorption capability. The procedure for generating the electrolyte film is straightforward, and the presence of abundant pores enables enhanced absorption of organic electrolytes, thereby augmenting the overall electrochemical performance. The results show that lithium metal batteries assembled using this GPE exhibit good rate and cycle stability performance. Therefore, the electrolyte synthesized by this cost-effective, efficient, and simple strategy has a promising practical application, especially in the next generation of high-performance LMBs.

## 2. Results and Discussion

**Figure 1:**
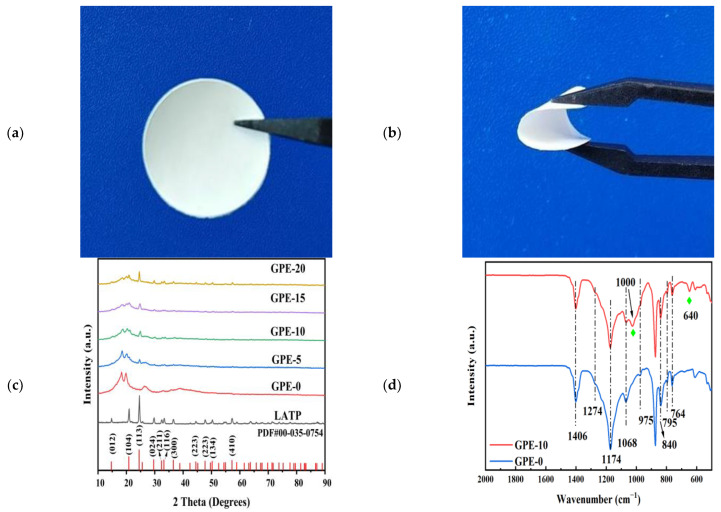
(**a**) Photographs of the GPE-10’s flat view; (**b**) photographs of the GPE-10’s bend diagram; (**c**) XRD patterns of PVDF-HFP incorporating varying amounts of LATP; (**d**) FT-IR patterns of GPE-10 and GPE-0.

Figure 1a,b is the photographs of GPE-10 in the plane and bent. It can be observed that GPE-10 can be easily bent and has good flexibility.

To determine the physical phases of PVDF-HFP and LATP, we obtained the XRD spectra in Figure 1c. The relative intensity and position of the diffraction peak of LATP powder are basically consistent with the standard XRD card (PDF#00-035-0754). Peaks at 14.7°, 20.8°, 24.5°, 29.6°, and 57.2° were located at the crystal surface (012), (104), (113), (024), and (410), indicating that the LATP powder was pure and well crystallized. The presence of both α- and β-phases of PVDF-HFP in the electrolyte film was confirmed by characteristic peaks detected around 18.3° and 19.8°.Characteristic peaks of LATP can be found in GPE-5, GPE-10, GPE-15, and GPE-20, indicating the presence of LATP particles in the GPE membrane. The intensity of the characteristic peaks of PVDF-HFP becomes smaller and broader with the increase in LATP content. The intensity of the β-phase increases relative to the intensity of the α-phase. This implies that the introduction of LATP perturbs the polymer–polymer interactions, resulting in a reduction in the crystallinity of PVDF-HFP polymers. Previous findings have illustrated that expanding the amorphous domain enhances lithium ions’ mobility and electrical conductivity [22]. The F atoms in the β-phase are attached to both sides of the polymer backbone in a trans-configuration, and the strong affinity between the polar functional group C-F and the lithium ions allows the lithium ions to be uniformly distributed at the lithium–metal interface, which can effectively inhibit the formation of lithium dendrites. In addition, this strong affinity can also make lithium ions evenly distributed in the GPE, providing a preferential diffusion path for lithium ions, which is conducive to the rapid charging and discharging of the battery [23].

Figure 1d exhibits the FTIR spectra of the polymer electrolyte, which provides additional insight into the interplay between PVDF-HFP and LATP. The peaks at 1406 cm^−1^ are characteristic signatures of C-F, and the vibrational band at 1174 cm^−1^ corresponds to the symmetric stretching of -CF_2_- [24]. Characteristic peaks at 764, 795, and 975 cm^−1^ represent CF_2_ bending and backbone bending, CH_2_ rocking, and CH_2_ twisting, all of them belonging to the α-phase crystals, and the characteristic absorption peaks at 840 (-CF_2_-symmetric) and 1274 cm^−1^ (C-C asymmetric stretching skeleton deformation) correspond to the polar β-phase [25]. The broad band of P-O stretching vibrations is located near 1000 cm^−1^, and O-P-O bending vibrations are located around 640 cm^−1^ [26,27]. These are the characteristic peaks of LATP. After the addition of LATP, the characteristic peaks of LATP were detected on the GPE-10FTIR absorption spectrum, and the characteristic peaks of PVDF-HFP persisted without appearing or disappearing, indicating the physical mixing of LATP and PVDF-HFP without any identifiable chemical transformation.

**Figure 2:**
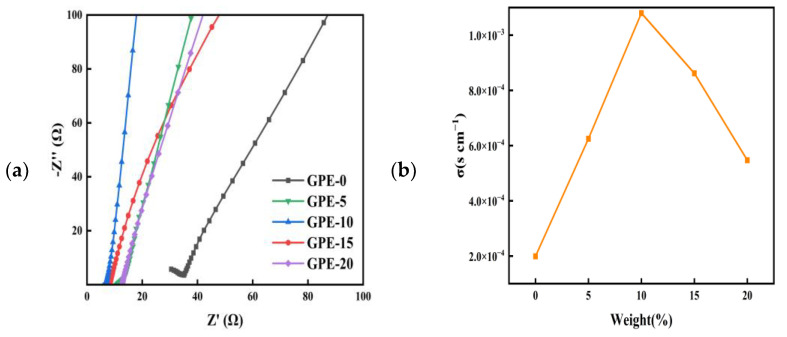

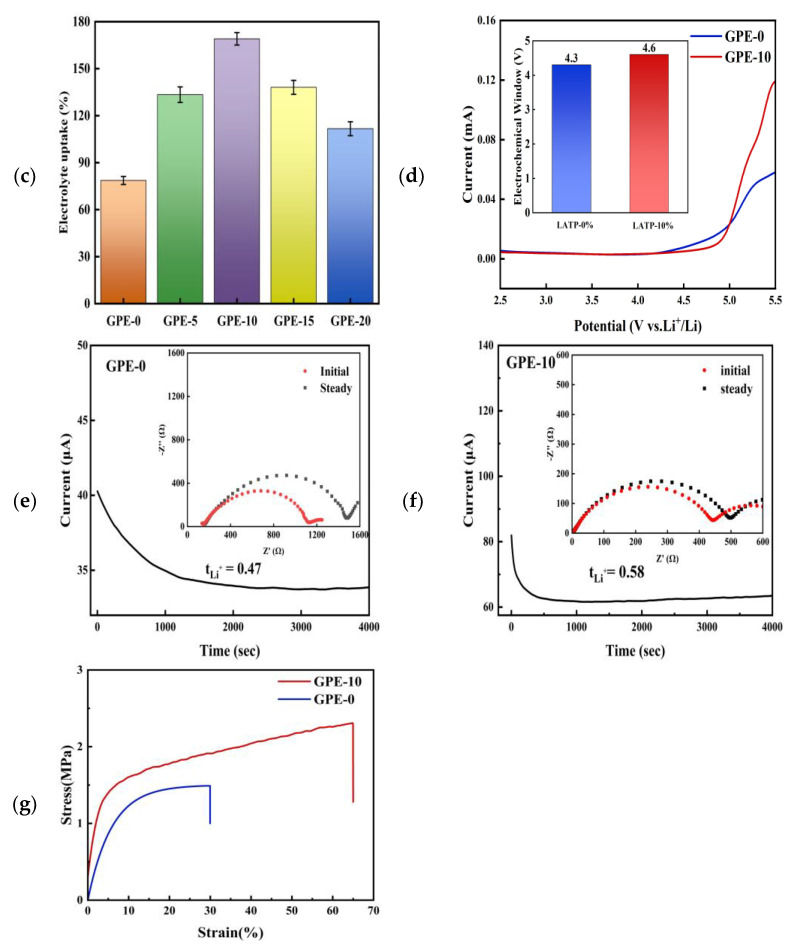
(**a**) Nyquist plot of GPE; (**b**) ionic conductivity of GPE; (**c**) electrolyte absorptivity of GPE; (**d**) LSV curves of GPE-0 and GPE-10; (**e**) polarization curve and impedance diagram of the cell before and after polarization (the inset) for GPE-0; (**f**) polarization curve and impedance diagram of the cell before and after polarization (the inset) for GPE-10; (**g**) stress-strain curves of GPE-0 and GPE-10.

Given its critical significance in battery performance, ionic conductivity measurement is imperative when examining the impact of varying LATP quantities on GPE. As can be seen from Figure 2a, each Nyquist plot consists of a semicircle at high frequencies and a slant line at low frequencies. The high-frequency semicircle represents the electrical conductivity in the block and within the crystal in GPE. The low-frequency tilt line is related to lithium-ion diffusion [28]. The abscissa of the junction between the low-frequency and high-frequency parts in the diagram is the volume resistance. The ionic conductivity is calculated from Equation (2), where R is read directly from the real part of the *X*-axis in the Nyquist plot, also at the junction of the semicircle and the inclined lithium-ion diffusion line. At 25℃, the volume impedance of GPE-10 is the smallest, and the volume impedance of GPE-0 is the largest. In the presence of well-dispersed LATP in the PVDF-HFP hybrid network, the crystallinity of the polymer electrolyte is lower and the resistance is reduced. Figure 2b showcases the Nyquist plots representing the samples with varying additions of LATP. The ionic conductivity of each sample was determined using Equation (2) for calculation. The ionic conductivities of GPE-0, GPE-5, GPE-10, GPE-15, and GPE-20 were ~2.0, 8.6, 10.8, 6.3, and 5.5 × 10^−4^ s cm^−1^, respectively, at 25 °C. The incorporation of LATP augmented the ionic conductivity of the electrolyte membrane. This enhancement is attributable to the intensified site-blocking influence exerted by the inorganic filler, LATP, which promotes the expansion of the amorphous phase within PVDF-HFP [29]. Consequently, the increased disorder and freedom of the organic chains facilitate a higher level of ionic conductivity within the electrolyte. Nonetheless, when the LATP content was increased to 20%, the high LATP content hindered the free movement of ions, resulting in a significant decrease in ionic conductivity [30,31]. 

The results of the liquid absorption test for GPE with different LATP contents are plotted in Figure 2c. The liquid electrolyte uptake of samples with different LATP content was 79.7%, 133.7%, 168.6%, 138.2% and 111.5%, respectively. The liquid uptake rate of GPE increased after the addition of LATP, and GPE could absorb more liquid electrolytes thus having a higher ionic conductivity. GPE-10 was chosen for subsequent studies because of its superior ionic conductivity and liquid absorption rate.

The electrochemical window of GPE-0 and GPE-10 was investigated using the linear voltammetric scanning (LSV) method. Figure 2d shows the LSV curves of GPE-0 and GPE-10. The LSV curve of GPE-10 is relatively smooth below 4.6 V and shows an increasing trend after 4.6 V, indicating that the electrolyte has undergone an oxidation reaction. The electrochemical window of GPE-10 (4.6 V) is superior to that of GPE-0 (4.3 V), and its electrochemical stability matches that of the LFP anode. 

The lithium-ion transfer number (t_Li+_) is particularly important for the performance of GPE. A high t_Li+_ electrolyte is conducive to promoting the migration of free lithium ions, thereby reducing the concentration polarization of cations/anions in the electrolyte so that the battery can be quickly charged and discharged. In contrast, electrolytes with lower t_Li+_ are prone to uneven dendritic lithium deposition, which will affect the battery’s ratio and cycle performance [32]. The timing current curves and AC impedance spectra of GPE-0 and GPE-10 are shown in Figure 2e,f. It is calculated that the t_Li+_ of GPE-10 is 0.58 and that of GPE-0 is 0.47. The addition of LATP greatly improves the migration times of lithium ions. On the one hand, LATP, as an inorganic solid electrolyte, has a high tLi+, which can be added to the solid electrolyte to provide additional transport paths for lithium ions. On the other hand, the larger surface area of LATP reduces the crystallinity of polymer electrolytes, thereby increasing the volume of the amorphous region and local segment motion. The resulting increase in the number of free lithium ions results in a higher t_Li+_ of GPE-10 [33,34]. At the same time, LATP has a high dielectric constant, which may affect ion transport in the polymer matrix because the local electric field at the interface between the polymer and the filler is changed. The high local field is conducive to the dissociation of lithium salts and the increase in ion mobility [35].

In addition, the mechanical stability of GPE was also studied. The tensile and deformation properties of GPE-10 are better than those of GPE-0 (Figure 2g). The stress–strain curve exhibits typical logarithmic behavior until the maximum attainable stress is reached, and no further elongation is observed after this critical level is reached, confirming the fracture of the sample. In GPE-10, the mechanical properties are expected to be improved due to the entanglement of the active filler LATP with the polymer chain. The stress and deformation were increased from 1.4 MPa to 2.3 MPa and 30% to 64%, respectively. The GPE-10 designed in this study exhibits a high degree of structural flexibility under loading conditions (i.e., stretching and relaxation) thanks to the high degree of flexibility of the PVDF-HFP network. The results show that the addition of LATP can improve the mechanical strength of PVDF-HFP and inhibit the penetration of Li dendrites.

**Figure 3:**
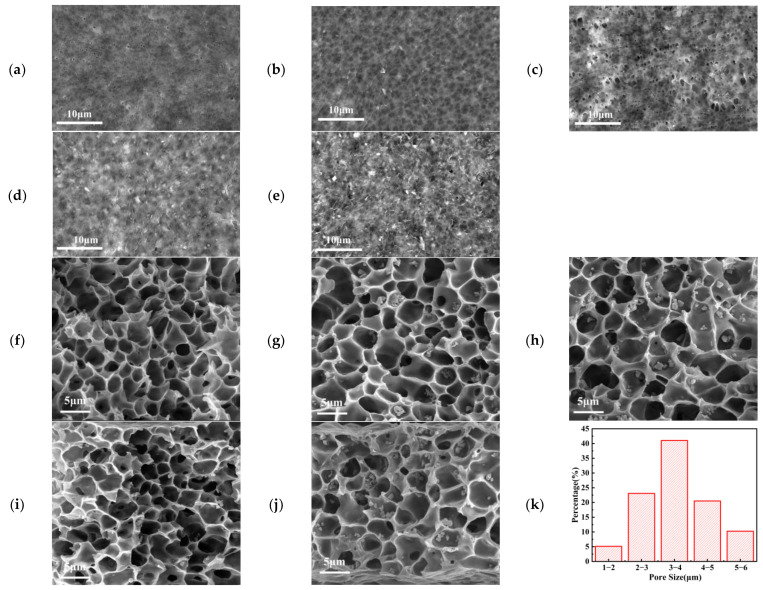
(**a**–**e**) SEM surface images from GPE-0 to GPE-20; (**f**–**j**) the SEM cross-section from GPE-0 to GPE-20; (**k**) the aperture distribution diagram of GPE-10.

The morphology and structure of GPE films were studied by scanning electron microscopy (SEM), as shown in Figure 3. As can be seen from Figure 3b–e, LATP powder is embedded in the surface structure of GPE-5 to GPE-20 samples. When LATP is not added and the content of LATP is small, the surface of the GPE film is smooth. With the increase in LATP content (such as GPE-20), the surface topography of the GPE film becomes rough, which may affect the interface contact between the electrolyte film and the positive and negative electrodes. At the same time, there are obvious holes on the surface of the GPE-10 membrane, which is conducive to the storage of electrolytes. The scanning electron microscope (SEM) images of each proportion of GPE are shown in Figure 3f–j. Each GPE film has a porous honeycomb structure. The morphology of the honeycomb pore structure suggests that liquid–liquid demixing dominates the initiation phase of the precipitation process. The polymer-rich phase matrix surrounding the honeycomb pores can be crystallized to form pore walls after liquid–liquid separation. This porous honeycomb structure is related to the preparation process using the immersion precipitation method, which can significantly improve the porosity and liquid absorption rate of the diaphragm [36]. The pore size of the GPE-10 membrane is the largest and deepest, with the pore size of GPE-10 ranging from 1 to 6 μm (Figure 3k). The pore sizes of GPE-15 and GPE-5 films were the second largest. GPE-0 and GPE-20 have the smallest and most shallow apertures. Larger and deeper holes can store more electrolyte, which corresponds to the suction rate described above. 

**Figure 4:**
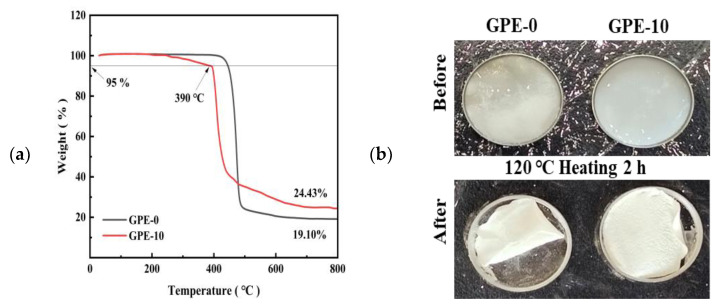
(**a**) TGA curves of GPE-0 and GPE-10; (**b**) comparison graphs of GPE-0 and GPE-10 before and after heating at 120 °C for 2 h.

Thermal stability is a very important parameter for GPE films, and the thermal stability of GPE-0 and GPE-10 is characterized by TGA curves (Figure 4a). The weight of GPE-0 decreases significantly in the temperature range of 400 to 500 °C. GPE-10 shows a significant weight loss at about 390 °C [37]. The observed phenomenon can be attributed to the breakdown of the electrolyte film. The temperature at which weight loss reaches 5% is generally considered to be the temperature of thermal decomposition. Therefore, the thermal decomposition temperature of GPE-10 is approximately 390 °C. The addition of LATP increases the amorphous portion of the polymer, which decreases the thermal stability of the GPE film. Although the thermal breakdown temperature of GPE-10 is lower than that of the GPE-0 electrolyte, the difference between the two is comparable. The decomposition temperature of LE is about 65 °C [30]. The thermal stability temperature of GPE-10 is higher than that of liquid electrolytes. This suggests that GPEs prepared using this scheme have better thermal stability than liquid electrolytes and can be operated at higher temperatures. In addition, the GPE-0 and GPE-10 membranes were compared after heating at 120 °C for 2 h as shown in Figure 4b. The GPE-0 electrolyte film shows obvious wrinkling after heating for 2 h, which means that the dimensional stability is very poor and may pose a serious threat to the safety of the battery, while only a small part of the right side of the GPE-10 electrolyte film undergoes wrinkling, and these results clearly show that the GPE-10 film has excellent thermal stability, which is conducive to the improvement of the safety of the battery in high-temperature applications.

**Figure 5:**
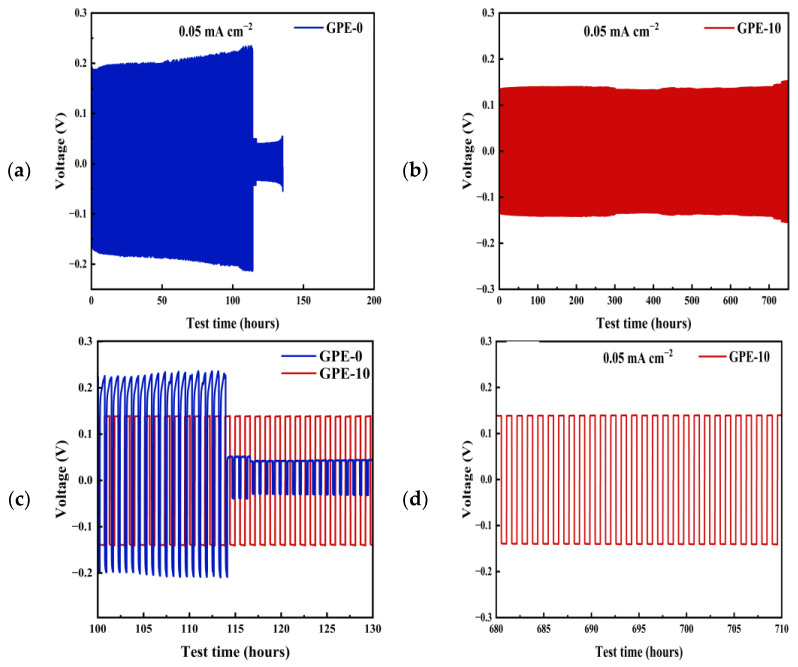
Electrochemical performance: (**a**) 0.05 mA cm^−2^ Li|GPE-0|Li symmetric cells; (**b**) 0.05 mA cm^−2^ Li|GPE-10|Li symmetric cells; (**c**) voltage-time plots of symmetric cells at 100–130 h; (**d**) voltage-time plots of symmetric cells at 680–710 h.

Voltage distribution plots of Li|GPE-0|Li and Li|GPE-10|Li symmetric cells’ plating/stripping with lithium cycling are given in Figure 5 to investigate the stability of the GPE-0 and GPE-10 interfaces at the lithium anode. The current density of the cell is 0.05 mA cm^−2^ and the charge/discharge time in each cycle is 0.5 h. The cell of Li|GPE-0|Li was short-circuited after 115 h. In contrast, the GPE-10 cell can be stably cycled for 750 h, and the polarization voltage does not fluctuate greatly. This shows that the gel electrolyte film containing 10 wt% LATP has good compatibility with lithium metal. The presence of a stable electrode–electrolyte interface effectively safeguards against short circuits and hinders the formation of lithium dendrites, considerably enhancing battery safety [38,39].

**Figure 6:**
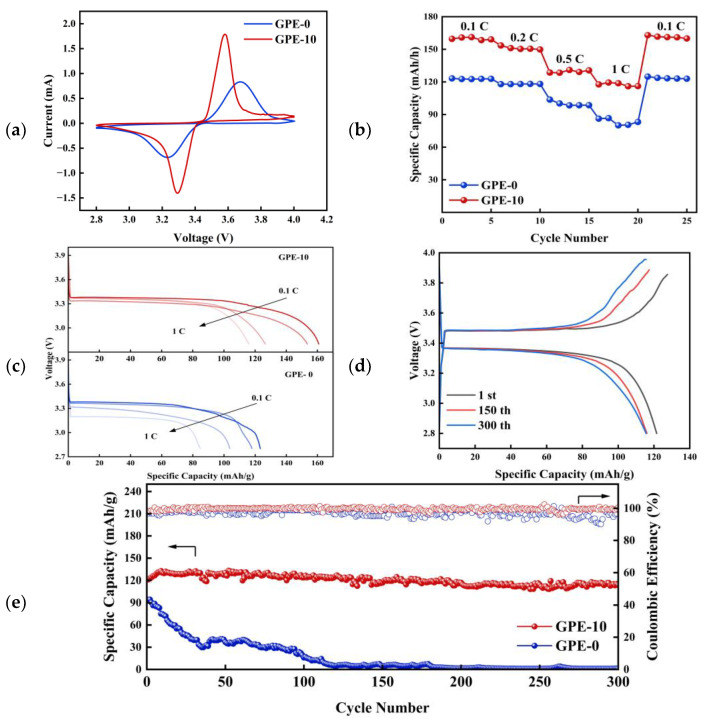
LFP|GPE-0|Li and LFP|GPE-10|Li full cell (**a**) CV curve graph, (**b**) cycle performance graph, (**c**) voltage-capacity profile at different rates, (**d**) voltage-capacity profile of LFP|GPE-10|Li full cell at 1st, 150^th^, and 300th cycle, and (**e**) cycling performance graph at 1C.

To demonstrate the practicality of GPE-10, we chose LFP as the cathode material to assemble the complete battery. The cyclic voltammetry curves of LFP|GPE-0|Li and LFP|GPE-10|Li cells at a scan rate of 0.2 mV/s are shown in Figure 6a. The area of the oxidation and reduction peaks is almost the same, which implies the reversibility of the lithiation/deoxidation process. The GPE-10 assembled cell possesses higher redox peaks, which indicates that the cell has a faster reaction rate and good conductivity. The reduced separation in the horizontal coordinates of the oxidation and reduction peaks observed in GPE-10 indicates a lesser degree of polarization relative to GPE-0 [40].

Figure 6b,c demonstrate the rate performance of GPE-0 and GPE-10 cells at different current densities. At various test rates ranging from 0.1C to 1C, the specific discharge capacities of LFP|GPE-10|Li cells exhibited values of 161.4, 153.5, 128.6, and 117.9 mAh/g, respectively. Upon reverting the testing rate back to 0.1C, the specific discharge capacity promptly reverts to 162.9 mAh/g. The specific discharge capacities of LiFePO_4_|GPE-0|Li cells were 123.3, 117.9, 103.7, and 86.7 mAh/g, respectively. Subsequent restoration of the current to 0.1C resulted in the recovery of the specific discharge capacity to 124.9 mAh/g. The discharge capacity of LFP|GPE-10|Li cells was always greater than that of LFP|GPE-0|Li cells at all test rates from 0.1 to 1C. LFP|GPE-10|Li cells demonstrate remarkable rate performance across various current densities, particularly under high-current conditions.

The LFP|GPE |Li cells were assembled to study the cycling stability of the cells under large magnification (Figure 6d,e). During the first cycle, the GPE-10 cell displayed a specific capacity of 121.5 mAh/g. Impressively, after 300 cycles, it maintained a high capacity retention rate of 94.0%, with a slight reduction in specific capacity to 114.2 mAh/g. In the inaugural cycle, the GPE-0 cell exhibited a specific capacity of 94.3 mAh/g, but after undergoing 300 cycles, the discharge capacity experienced a substantial decline to a mere 1.3 mAh/g. LFP|GPE-10|Li cells exhibit good rate capacity and cycling performance.

## 3. Conclusions

Gel polymer electrolyte PVDF-HFP/LATP with a honeycomb porous structure was prepared using the impregnation precipitation method. By comparing GPE-0 and GPE-10, it was found that the addition of 10% LATP could most effectively improve the physicochemical and electrochemical performances of the PVDF-HFP-type GPE. Compared with GPE-0, GPE-10 has an ionic conductivity of 1.03 × 10^−3^ S cm^−1^ and a lithium-ion transfer number of 0.56. In addition, it has an electrochemical window of 4.6 V and better interfacial stability. The initial discharge specific capacity of GPE-10 assembled into a battery was 121.5 mAh/g at 1C, and the capacity retention was 94.0% after 300 cycles. In addition, the rate performance of LiFePO_4_|GPE-10|Li is better. The cost-effective, efficient, and straightforward synthesis strategy used to prepare this electrolyte holds immense promise for its integration into next-generation lithium batteries, known for their high performance.

## 4. Materials and Methods

### 4.1. Materials

The raw materials include polyvinylidene fluoride-hexafluoropropylene (PVDF-HFP, Arkema, Colombes, France), Li_1.3_Al_0.3_Ti_1.7_(PO_4_)_3_ (LATP, Macklin, Shanghai, China), N, N-dimethylformamide (DMF, Macklin, Shanghai, China), LiFePO_4_ (LFP, Macklin, Shanghai, China), conductive carbon black (SUPER P, Macklin, Shanghai, China), and polyvinylidene fluoride (PVDF, Macklin, Shanghai, China).

### 4.2. Preparation of Gel Electrolyte

**Figure 7:**
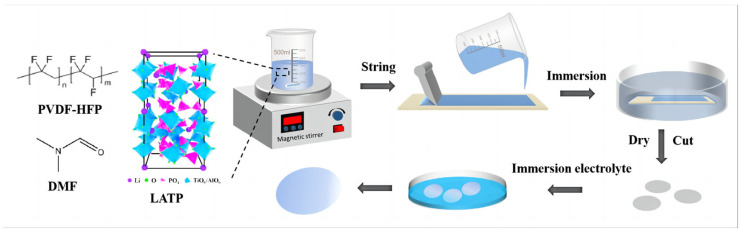
Schematic illustration of the formation process of GPE.

The production process is shown in Figure 7. In the solution of DMF, PVDF-HFP and LATP powder were mixed. The solution was heated to 45 °C and stirred for 4 h. Then, it was poured onto a PTFE sheet and evenly spread with a spatula. After scraping, the PTFE plate was immersed in deionized water. The formed porous membrane was then immersed in deionized water for 5 h to separate the remaining DMF solvent into the deionized water. The desolvated porous film was dried in a vacuum drying oven. The dried porous membrane was cut into φ16 mm. The GPE was immersed in an organic liquid electrolyte (1 mol L^−1^, LiPF_6_ in EC:DMC:EMC = 1:1:1 Vol% with 1% VC) for 0.5 h in an argon-filled glove box. To investigate the effect of different LATP contents on GPE, the contents of PVDF-HFP with LATP were selected as 100:0, 100:5, 100:10, 100:15, and 100:20 and named GPE—(0, 5, 10, 15, 20), respectively.

### 4.3. Production of LFP Positive Electrode

A certain mass of lithium iron phosphate and conductive carbon black was fully ground in the onyx mortar for 40 min, with the ground lithium iron phosphate, conductive SUPER P, and PVDF in accordance with the mass ratio of 8:1:1. Then, an appropriate amount of N-methyl pyrrolidone (NMP, Macklin, Shanghai, China) was added, and the mixture was stirred at room temperature for 4 h in the coating machine, and then transferred to a vacuum oven at 60 °C, where it was dried for 36 h. After drying, the poles were cut into 14 mm pieces with a tablet press and transferred to a glove box for later use.

### 4.4. Assembling the Battery

The CR2025 button cell was assembled in a glove box filled with argon gas, using the LFP electrode as the cathode and lithium metal as the anode. The procedure outlined was followed to assemble the battery.

### 4.5. Characterization of Materials

The prepared GPEs were analyzed by X-ray diffractometer (XRD, DX-2700, Dandong, China, Cu-Kα, 40 kV × 30 mA). The surface and cross-section of the GPE were examined utilizing a scanning electron microscope (SEM, Phenom spectra G2, Shanghai, China) for observation and analysis. Thermogravimetric analysis (TGA) was conducted in a nitrogen atmosphere using a TGA system (NetzschF3Tarsus, Bayern, Germany), wherein the temperature was ramped from 30 to 800 °C at a rate of 10 °C per minute. The determination of the functional groups in the GPEs prepared at room temperature was conducted using Fourier transform infrared spectroscopy (FTIR, Spectrum 100, PerkinElmer, Shelton, CT, Massachusetts, USA) within the range of 4000–400 cm^−1^.

The liquid absorption rates of different electrolyte membranes were calculated according to Equation (1).

(1) $$Electrolyte\;uptake=\left(M_{2}-M_{1}\right)/M_{1}\times 100\%$$

*M*_1_ and *M*_2_ represent the initial and final mass of the electrolyte film following immersion in n-butanol, respectively.

Stainless steel spacer (SS)|GPE|Li cells were assembled and the electrochemical window of the electrolyte film was tested by linear scanning voltammetry (LSV, DH7000, Donghua, Jingjiang, China) on an electrochemical workstation. The scanning interval was set to 2.5–5.5 V, the scanning speed was 0.5 mV/s, and the testing temperature was room temperature.

The assembled SS|GPE|SS symmetric cells were subjected to an impedance test at room temperature. The ionic conductivity was calculated by Equation (2). The frequency range was set from 1 to 10^6^ Hz. *σ* represents the ionic conductivity, and *L*, *S*, and *R* represent the thickness (cm), cross-sectional area (cm^2^), and the bulk resistance (Ω) of the electrolyte, respectively.

(2) $$\sigma =\frac{L}{R\cdot S}$$

The Li|GPE|Li symmetric cell was used for the lithium transference test at room temperature to calculate the lithium transference number using Equation (3), where the DC polarization voltage is denoted as Δ*V*, *I*_0,_ and *I*_SS_ denote the current before and after polarization, and *R*_0_ and *R*_ss_ denote the impedance of the electrolyte membrane before and after polarization, respectively.

(3) $$t_{Li}^{+}=\frac{I_{SS}\left(\Delta V-R_{0}I_{0}\right)}{I_{0}\left(\Delta V-R_{SS}I_{SS}\right)}$$

The assembled LFP|GPE|Li cells were charged and discharged using a battery test system to examine the charging and discharging curves, multiplication performance, and cycling performance of the assembled batteries.

## Data Availability

The original contributions presented in the study are included in the article, further inquiries can be directed to the corresponding authors.
